# Transcriptional Regulation of gga-miR-451 by AhR:Arnt in *Mycoplasma gallisepticum* (HS Strain) Infection

**DOI:** 10.3390/ijms20123087

**Published:** 2019-06-24

**Authors:** Yabo Zhao, Yali Fu, Yingfei Sun, Mengyun Zou, Xiuli Peng

**Affiliations:** Key Laboratory of Agricultural Animal Genetics, Breeding and Reproduction, Ministry of Education; College of Animal Science and Technology and College of Veterinary Medicine, Huazhong Agricultural University, Wuhan 430070, China; zyb@webmail.hzau.edu.cn (Y.Z.); fuyali@webmail.hzau.edu.cn (Y.F.); sunyingfei@webmail.hzau.edu.cn (Y.S.); zoumengyun@webmail.hzau.edu.cn (M.Z.)

**Keywords:** AhR:Arnt, gga-miR-451, *Mycoplasma gallisepticum* HS strain, YWHAZ

## Abstract

MicroRNAs (miRNAs) have been determined to be important regulators for pathogenic microorganism infection. However, it is largely unclear how miRNAs are triggered during pathogen infection. We previously reported that the up-regulation of gga-miR-451 negatively regulates the *Mycoplasma gallisepticum* (MG)-induced production of inflammatory cytokines via targeting tyrosine3-monooxygenase/tryptophan5-monooxygenase activation protein zeta (YWHAZ). The aim of this study was to investigate the mechanism regulating gga-miR-451 in MG infection in chickens. Analysis of gga-miR-451 precursor, pri-miR-451, and pre-miR-451 indicated that the regulation occurred transcriptionally. We also identified the transcriptional regulatory region of gga-miR-451 that contained consensus-binding motif for aryl hydrocarbon receptor (AhR) and aryl hydrocarbon receptor nuclear translocator (Arnt) complex, which is known as the transcription factor that regulates gene expression. Luciferase reporter assays combined with chromatin immunoprecipitation (ChIP) demonstrated that AhR:Arnt bound directly to the promoter elements of gga-miR-451, which were responsible for gga-miR-451 transcription in the context of MG infection. Furthermore, upregulation of AhR:Arnt significantly induced gga-miR-451 and inhibited YWHAZ expression, suggesting that AhR:Arnt may play an anti-inflammatory role in MG infection. This discovery suggests that induced gga-miR-451 expression is modulated by AhR:Arnt in response to MG infection.

## 1. Introduction

MicroRNAs (miRNAs) are composed of a large class of post-transcriptional regulators of gene expression, and the majority of miRNAs are originally transcribed in the nucleus RNA polymerase II. Following transcription, the primary transcripts of miRNAs (pri-miRNAs) are processed by RNAase III enzyme, Drosha, into ~65–100 nucleotide (nt) precursor miRNAs (pre-miRNAs) to be transported into the cytoplasm and cleaved by the RNAase III, Dicer, to give rise to double-stranded, mature miRNAs (~22 nt). The miRNA passenger strand is degraded while the miRNA guide strand is loaded into an Argonaute protein to form the RNA-induced silencing complex (RISC). The RISC then produces mature miRNA, which enables partial base pairing and negative regulation of protein synthesis and/or mRNA degradation in the majority of cases [1]. In chickens, miRNAs have been reported to regulate diverse developmental, physiological, and pathological processes including avian diseases [2]. For example, gga-miR-19a activates the NF-κB signaling pathway through targeting ZMYND11 and promotes the proliferation of chicken fibroblast (DF-1) cells upon *Mycoplasma gallisepticum* HS strain (MG-HS) infection [3]. gga-miR-23b promotes the replication of Avian Leukosis virus by repressing the expression of IRF1 [4]. gga-miR-375 inhibits cell proliferation in tumorigenesis post subgroup J avian leukosis virus infection [5]. Although the function of host miRNAs has been generally studied during various foreign pathogen infections, there is very limited knowledge on how the expression of miRNAs is triggered during these diseases.

Approximately 50%–70% of miRNAs are located in intergenic sites or in antisense orientation to annotated genes with independent transcription units. While the remaining miRNAs are encoded within the introns of protein coding genes, they might be transcribed in parallel with host transcripts and as part of the host genes [6]. Different mechanisms of biogenesis are required depending on the two different transcription classes of miRNA. Previous studies reported that the expression of miRNAs was generally regulated by transcriptional factors and co-regulators. For instance, the transcription factor *Ppara* directly binds the promoter region of Hsa-miR-181a2 and up-regulates its expression [7]. In mouse granulosa cells, transcription factor CP2 enhances the expression of miR-144 [8]. Several miRNAs, including let-7, miR-21, miR-146a, miR-155, miR-181, and the miR-17-92 cluster have been demonstrated as targets of the *NF-κB* transcription factors [9].

Recently, chronic respiratory disease (CRD) of chickens and turkeys has emerged as a great challenge to poultry farmers and creates a large financial burden for poultry industries around the world. The major causative agent is *Mycoplasma gallisepticum* (MG), which is a bacterium belonging to the class *Mollicutes* and the family *Mycoplasmataceae* and has the ability to penetrate chicken fibroblasts and HeLa cells to establish infection [10]. During infection, MG integrates with host respiratory epithelial cells (trachea, lungs, and air sacs) and causes local or systemic inflammatory response [11]. We previously reported that gga-miR-101, gga-miR-19a, and gga-miR-451 were up-regulated in both MG-infected DF-1 cells and MG-infected chicken embryonic lungs to modulate MG-triggered inflammation by targeting genes involved in inflammatory signal pathways [3,12,13]. However, the knowledge of the mechanisms for how miRNA genes themselves are triggered has comparatively lagged behind.

Our previous study indicated the property of gga-miR-451 as an anti-inflammatory miRNA, which negatively regulates the MG-induced production of inflammatory cytokines through targeting YWHAZ [13]. In mammals, miR-451 has also been reported to play a critical role in regulating inflammation in influenza-infected cells [14]. To further explain the effects of gga-miR-451 in MG-HS infection, it will be crucial to understand how gga-miR-451 gene is up-regulated. In this study, we found that the pri-miR-451 and pre-miR-451 were up-regulated in MG-HS infection, suggesting that regulation of gga-miR-451 occurs transcriptionally. Promoter analysis revealed that AhR:Arnt plays an important role in induction of gga-miR-451 transcription upon MG-HS infection. Furthermore, we found that AhR:Arnt was significantly up-regulated upon MG-infected DF-1 cells and was a negative regulator of YWHAZ expression. To our knowledge, this is the first time that the transcriptional regulation of gga-miR-451 was identified.

## 2. Results

### 2.1. Primary Transcript of gga-miR-451 Is Induced in MG-infected DF-1 Cells

**Figure 1:**
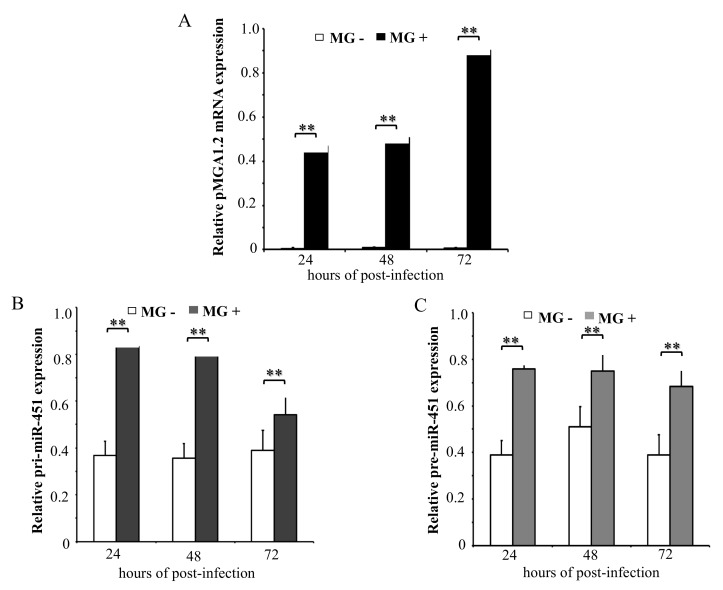
Differential expression of primary transcripts of *Mycoplasma gallisepticum* (MG)-induced gga-miR-451. The total RNA was extracted at 24, 48, and 72 h post-infection (hpi), and the expression of pMGA1.2 (**A**), pri-miR-451 (**B**), and pre-miR-451 (**C**) were assessed by RT-qPCR normalizing to the expression of glyceraldehyde-3-phosphate dehydrogenase (GAPDH) in the samples. Three independent experiments, each with three replicates, were performed. Student’s *t* test was used to analyze the significant differences. The plotted data points show the means ± SDs, and the asterisks indicate statistically significant differences (** *p* < 0.01).

Our previous data show that gga-miR-451 level was significantly up-regulated in both MG-infected DF-1 cells and MG-infected chicken embryonic lungs [13]. To determine the kinetics of primary gga-miR-451 transcript (pri-miR-451) and gga-miR-451 precursor (pre-miR-451) after MG infection, DF-1 cells were infected with MG-HS for the indicated times, with non-infected acting as the control group. As shown in Figure 1A, pMGA1.2, the adhesion protein from MG-HS, was highly expressed at 24, 48, and 72 h post-infection (hpi). We further measured the expression of pri-miR-451 and pre-miR-451, and they were up-regulated with no time-dependent increase in cells following MG infection (Figure 1B,C). These data suggest that the upregulation of gga-miR-451 may happen at the transcriptional level.

### 2.2. Identification of the Promoter Region for gga-miR-451

**Figure 2:**
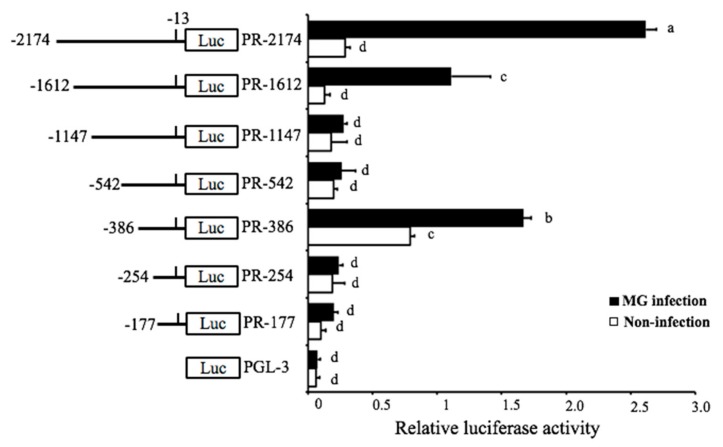
Identification of the boundary of gga-miR-451 promoter region. Chicken fibroblast (DF-1) cells were co-transfected with promoter vectors (100 ng) of different length of the gga-miR-451 promoter region and PRL-TK (10 ng) for 12 h and then either left uninfected or infected with 30 µL of MG-HS, At 24 hpi, samples were collected and the luciferase activity was measured by a dual-luciferase glow assay; the firefly luciferase activity was standardized to Renilla luciferase activity, and pGL3 was used as control. The data are expressed as the means ± SDs. Different lowercase letters represent *p* < 0.01, determined by ANOVA.

The detailed mechanism of how the MG-HS infection triggered the expression of pri-miR-451 was studied. The location and genomic information of gga-miR-451 that were acquired from NCBI (https://www.ncbi.nlm.nih.gov/gene/777915) showed that gga-miR-451 resides on chromosome 19 and between ERAL1 gene and FLOT2 gene. To examine the transcriptional regulation of gga-miR-451 in MG-infected DF-1 cells, we tried to determine the promoter region of gga-miR-451. To identify the range of the minimal promoter region (PR), an upstream region of about 2.2-kb DNA fragment was selected for promoter mapping, followed by a series of deletions of this DNA fragment and construction into luciferase reporter plasmids (pGL-3). These reporter plasmids were separately transfected into DF-1 cells to determine the basal and MG-induced promoter activities. The luciferase reporter assay indicated that full-length reporter (PR-2174) had explicit MG inducibility, which was reduced by three cuttings of this full-length region, PR-1612, PR-1147, and PR-542 (Figure 2). However, the reporter PR-386 had a higher luciferase activity in both non-infected DF-1 cells and MG-infected DF-1 cells, compared to the reporter PR-254, PR-177, suggesting that PR-386 (−386 bp to −254 bp, 132 bp) is required for gga-miR-451 transcriptional activity and possesses fully intact promoter activity.

### 2.3. AhR:Arnt Binding Sites are Necessary for gga-miR-451 Promoter Activity

**Figure 3:**
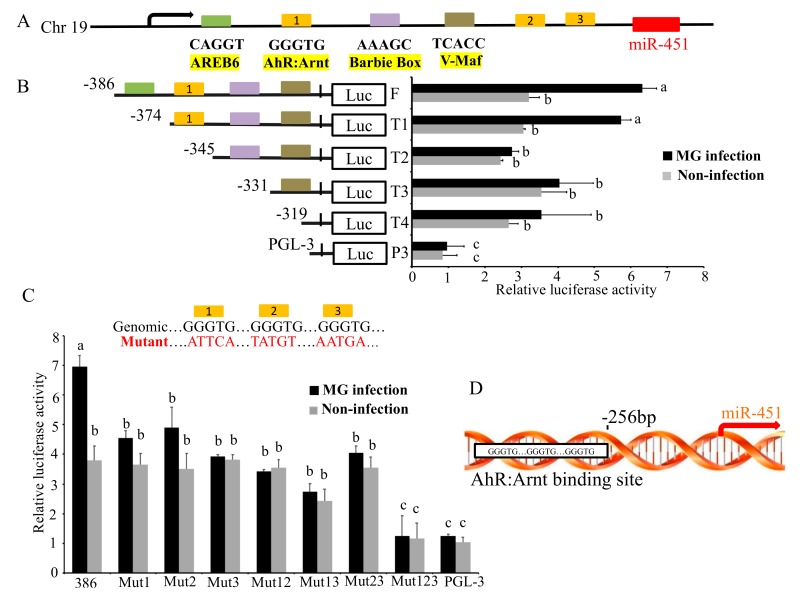
aryl hydrocarbon receptor (AhR):aryl hydrocarbon receptor nuclear translocator (Arnt) regulates gga-miR-451 promoter activity. (**A**) Schematic diagram of gga-miR-451 genomic loci on chicken chromosomes 19. Potential binding sites of AREB6, AhR:Arnt, Barbie Box, and v-Maf transcriptional factors (TFs) are shown as different color boxes; the sequences are under the boxes. (**B**) Schematic representation of truncation T1 to T4 in the full-length gga-miR-451 promoter. DF-1 cells were co-transfected with promoter vectors (100 ng) with different deletions and PRL-TK (10 ng) for 12 h and then either uninfected or infected with 30 µL of MG-HS. At 24 h post-infection, both firefly and renilla luciferase activity were measured through a dual-luciferase reported assay. (**C**) Schematic representation of different point mutations (Mut1, 2, and 3) in the wild-type promoter (386). The details of dual-luciferase reported assay are the same as above. (**D**) Sequence schematic diagram of AhR:Arnt binding site in the promoter region of gga-miR-451. The data are shown as means ± SDs. Different lowercase letters represent *p* < 0.01, determined by ANOVA.

To further decipher the transcription factors binding to the core promoter of gga-miR-451, we used the transcription factor prediction software from gene-regulation (http://gene-regulation.com) and JASPAR (http://jaspar.genereg.net) to analyze the putative binding sequences (−386 bp to −254 bp), the potential transcription factors, including AREB6, AhR:Arnt, Barbie Box, and v-Maf predicted within gga-miR-451 promoter (Figure S1). The transcription factor-binding sites (TFBS) were also identified in the PR-386 region (132bp) by the prediction software with three binding sites by AhR:Arnt (Figure 3A). To detect if the predicted transcription factors were responsive to gga-miR-451 transcription, we constructed dual-luciferase reporter plasmids carrying a series of truncated promoters, including full-length (F), truncation 1 (T1-374bp), T2-345bp, T3-331bp, and T4-319bp (Figure 3B). The luciferase activity assay showed that deletion of AREB6 binding site did not have significant effect on luciferase activity compared to the full-length promoter construction. However, when AhR:Arnt binding site was truncated, the MG-induced luciferase activity was significantly decreased (Figure 3B), suggesting AhR:Arnt binding site was necessary for gga-miR-451 promoter activation. Furthermore, deletion of Barbie Box and v-Maf binding sites had no significant effect on luciferase activity compared to the deletion of AhR:Arnt binding site. To further clarify whether the three AhR:Arnt binding sites were all core sites, seven different dual-luciferase reporter plasmids including mutant 1 (Mut1), Mut2, Mut3, mutants 1 and 2 (Mut12), Mut13, Mut23, and Mut123, were constructed. As shown in Figure 3C, mutant123 significantly affected MG-induced luciferase activity compared to the full-length promoter construction, whereas the other different mutants did not affect non-infection luciferase activity but reduced MG-induced promoter activity. These results indicate that the three AhR:Arnt binding sites are necessary for the activation of gga-miR-451 promoter by MG infection (Figure 3D).

### 2.4. AhR:Arnt Enhances the Transcriptional Activity of the gga-miR-451 Promoter

**Figure 4:**
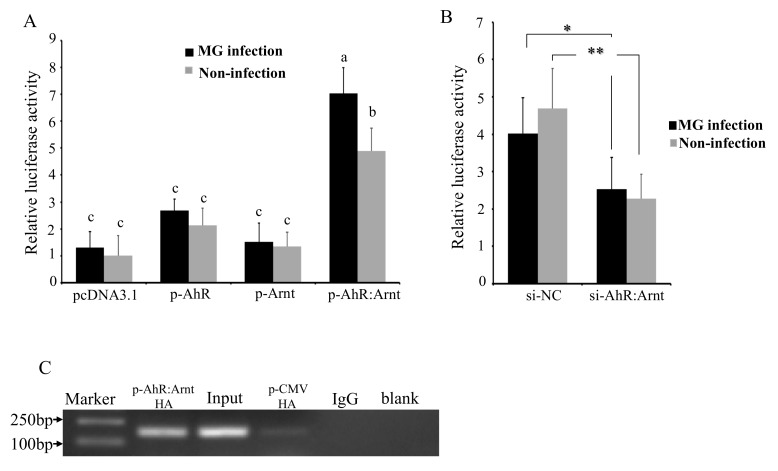
AhR:Arnt directly binds to gga-miR-451 promoter. (**A**) DF-1 cells were co-transfected with pGL3-386 vector (100 ng), PRL-TK vector (10 ng), and pcDNA3.1, p-AhR, p-Arnt, or p-AhR:Arnt (50 ng AhR and 50 ng Arnt) for 12 h, then either left uninfected or infected with MG. At 24 hpi, luciferase reporter assays detected the gga-miR-451 promoter activities. (**B**) DF-1 cells were co-transfected with pGL3-386 vector, PRL-TK vector, and si-NC, si-AhR:Arnt for 12 h, then either left uninfected or infected with MG-HS. At 24 hpi, luciferase reporter assays detected the gga-miR-451 promoter activities. (**C**) ChIP-qPCR assay was performed using chromatin isolated from pCMV-C-HA-AhR:Arnt/pCMV-C-HA-transfected DF-1 cells. Anti-HA was used for immunoprecipitation of the chromatin DNA fragment, the precipitated DNA was extracted and amplified by qPCR using primers spanning the AhR:Arnt binding sites. IgG was used as negative control, the input (total DNA extract) was used as positive qPCR control. The ultrapure water was used instead of the template as blank (three replicates were performed). The data are shown as means ± SDs. Different lowercase letters represent *p* < 0.01, ** *p* < 0.01, * *p* < 0.05, determined by ANOVA.

To verify the role that AhR:Arnt plays in MG-induced activation of gga-miR-451 promoter, the effects of AhR:Arnt on the transcriptional activity of the gga-miR-451 promoter were evaluated by over-expressing AhR:Arnt with pcDNA3.1 vector and silencing AhR:Arnt with siRNA. pcDNA3.1-AhR (p-AhR) and pcDNA3.1-Arnt (p-Arnt) could substantially increase AhR and Arnt expression. The siRNA-AhR:Arnt (si-AhR:Arnt) could significantly inhibit AhR:Arnt expression in DF-1 cells (Figure S2). The p-AhR, p-Arnt, pcDNA3.1-AhR:Arnt (p-AhR:Arnt), control pcDNA3.1, si-AhR:Arnt, or a nonspecific control siRNA (si-NC) was co-transfected with the pGL3-386 vector into DF-1 cells, and then either left uninfected or infected with MG at 24 hpi, followed by luciferase reporter assays. As shown in Figure 4A,B, overexpression of AhR:Arnt together significantly increased MG-induced gga-miR-451 promoter activity. However, overexpression of AhR or Arnt on their own did not affect the luciferase activity. In contrast, knockdown of AhR:Arnt decreased gga-miR-451 promoter activity. These results indicate that AhR:Arnt regulates gga-miR-451 promoter activity.

To further confirm that AhR:Arnt can regulate the activity of the core promoter of gga-miR-451, ChIP assay was performed to analyze whether AhR:Arnt binds to the gga-miR-451 promoter. We constructed pCMV-C-HA-AhR and pCMV-C-HA-Arnt, transfected the two vectors or empty vector (pCMV-V-HA) into DF-1 cells, and isolated chromatin for immunoprecipitation. qPCR analysis showed that a 141-bp DNA region was amplified from the anti-HA (pCMV-C-HA-AhR:Arnt) precipitates in DF-1 cells, whereas the DNA fragment was not amplified from anti-HA (pCMV-C-HA), the anti-IgG, and Mock precipitates (Figure 4C). Taken together, these results indicate that AhR:Arnt specifically binds to the gga-miR-451 promoter region and are important transcriptional regulators of gga-miR-451.

### 2.5. AhR:Arnt Regulates gga-miR-451 and YWHAZ Expression

**Figure 5:**
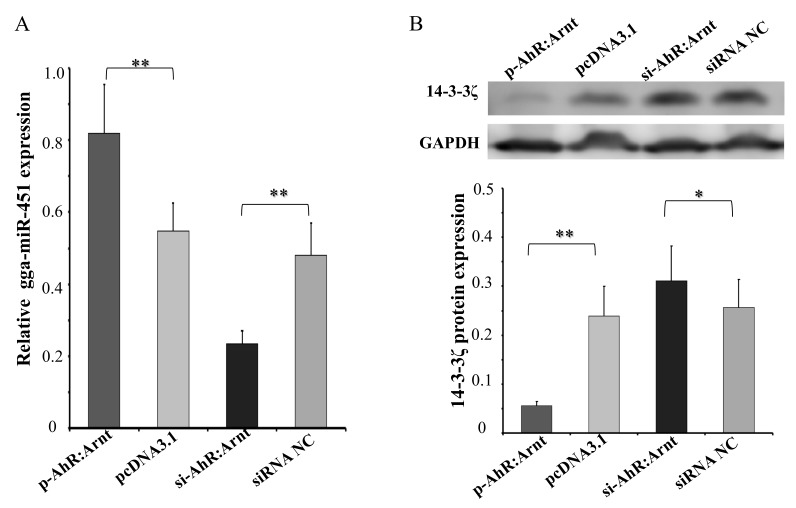
AhR:Arnt promotes gga-miR-451 expression decreases the expression of tyrosine3-monooxygenase/tryptophan5-monooxygenase activation protein zeta (YWHAZ). (**A**) qRT-PCR was used to detect gga-miR-451 level 48 h after DF-1 cells were transfected with p-AhR:Arnt, pcDNA3.1, si-AhR:Arnt, or siRNA NC. (**B**) Western blot analysis was used to detect YWHAZ protein level 48 h post transfection with p-AhR:Arnt, pcDNA3.1, si-AhR:Arnt, or siRNA NC. The results are expressed as the means ± SDs, with three independent replicates per group. (* *p* < 0.05, ** *p* < 0.01).

To confirm that the AhR:Arnt could regulate gga-miR-451 expression, we transfected pcDNA3.1-AhR:Arnt (p-AhR:Arnt), pcDNA3.1, siRNA-AhR:Arnt (si-AhR:Arnt), or siRNA NC into DF-1 cells, respectively. By using qRT-PCR, AhR:Arnt overexpression significantly increased gga-miR-451 expression, whereas knockdown of AhR:Arnt decreased gga-miR-451 expression (Figure 5A). In addition, we recently reported that YWHAZ is a target gene of gga-miR-451 with negative regulation in DF-1 cells [13]. We hypothesize that AhR:Arnt can affect YWHAZ expression through regulating gga-miR-451. To test this hypothesis, Western blot analysis of identical transfected samples was performed (Figure 5B). P-AhR:Arnt markedly suppressed endogenous YWHAZ protein level. In contrast, si-AhR:Arnt promoted YWHAZ expression. These data suggest that AhR:Arnt binds to the core promoter of gga-miR-451, induces the expression of mature gga-miR-451, and eventually suppresses YWHAZ expression.

### 2.6. MG Infection Significantly Upregulates AhR:Arnt Expression

**Figure 6:**
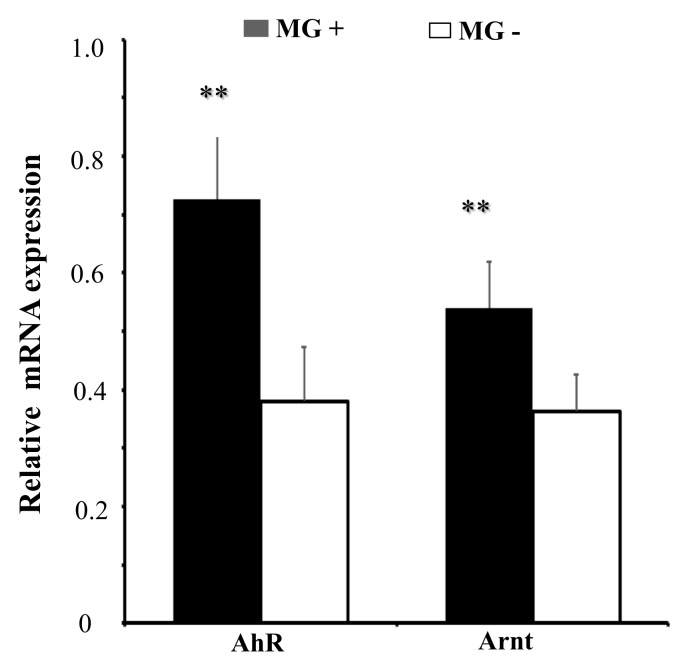
MG infection upregulates the expression of AhR and Arnt. DF-1 cells were infected with MG-HS as described in Materials and Methods and the total RNA was extracted. Expression of mRNA of AhR and Arnt was assessed by RT-qPCR normalized to GAPDH. The results are expressed as the means ± SDs, with three independent replicates per group. (** *p* < 0.01).

To further explore the role of AhR:Arnt upon MG-HS infection, we measured the expression of AhR:Arnt in MG-HS-infected DF-1 cells. RT-qPCR results showed that AhR and Arnt were significantly up-regulated in MG-infected DF-1 cells relative to its expression in non-infected DF-1 cells (Figure 6). These results suggest that AhR:Arnt plays an important role in MG infection.

## 3. Discussion

We found that miR-451 is highly conserved in the majority of vertebrate and is widely expressed in different tissues [15]. It has been found to be an important regulator of inflammation and immune responses in mammals. For example, miR-451 suppresses CD4^+^ T cell proliferative to *Plasmodium* parasite responses and miR-451−/− mice increased immune responses to infection [16]. Inhibition of miR-451 expression is correlated with increases in the secretion of IL-6, TNF, CCL5/RANTES, and CCL3/MIP1α in mice dendritic cell [14]. In addition, miR-451 inhibits the expression of NF-κB-mediated proinflammatory molecules via inhibiting LMP7 in diabetic nephropathy [17]. We previously reported that gga-miR-451 is up-regulated in MG-infected DF-1 cells and chicken embryonic lungs with potential anti-inflammatory properties through targeting YWHAZ. gga-miR-451 inhibits the cell proliferation and cell cycle progression, and promotes cell apoptosis to facilitate MG replication [13]. Due to the important role of gga-miR-451 in MG infection, the transcriptional level regulation of the gga-miR-451 has received extensive concerns.

The biogenesis of miRNAs is a multistep and sophisticated process. They are initially transcribed into pri-miRNAs, then pri-miRNAs are processed into pre-miRNAs, and finally, into mature miRNAs [1]. The mechanism of gga-miR-451 upregulation by MG infection was studied in this article. We found the upregulation of pri-miR-451 and pre-miR-451 in MG-infected DF-1 cells, indicating that the regulation of gga-miR-451 involves transcriptional mechanisms. Interestingly, the expression of pMGA1.2 at 72 h post-infection was higher than its expression at 24 or 48 hpi. But pri-miR-451 and pre-miR-451 did not follow this similar trend. This inconsistency may be due to the other complicated post-transcriptional regulation of gga-miR-451 [18]. Thus, although these data demonstrate that MG-induced gga-miR-451 was regulated transcriptionally, there remains a possibility that expression of gga-miR-451 may also be regulated at the processing level.

Most miRNAs are initially transcribed in the nucleus. Transcription factors can bind to the promoters or enhancers of miRNAs and regulate their expression. Then, miRNAs regulate the subsequent gene expression at the post-transcriptional level [19]. This may be an important element of host responses against pathogenic microorganisms infection [20]. In this study, the promoter region of gga-miR-451 gene was analyzed by the luciferase reporter assay. The data showed that the reporters PR-2174, PR-1612, and PR-386 had both foundational promoter activity and MG inducibility, but other reporters could not drive MG-induced promoter activity. It is possible that the region from −1147 to −386 may contain transcriptional repressors inhibiting MG-induced luciferase activity, whereas the region from −2174 to −1147 may contain transcriptional activators, which weaken repressors that improve MG-induced luciferase activity. However, the underlying mechanism remains to be clarified in future studies. Furthermore, functional assays of gga-miR-451 promoter revealed that transcription factor AhR:Arnt is at least partially responsible for the transcription of gga-miR-451 through three of the AhR:Arnt binding sites in DF-1 cells following MG infection. Mutant123 significantly decreased the luciferase activity of both MG-infection and non-infection compared to the full-length promoter construction (Figure 3C), whereas PR-345 did not cause decreased expression in non-infection (Figure 3B). It is possible that the PR-345 region still had two binding sites working. Direct binding of AhR:Arnt to the promoter of gga-miR-451 gene was further identified by ChIP assay. Therefore, AhR:Arnt-mediated transcriptional processing of gga-miR-451 may contribute to the upregulation by MG.

AhR is an evolutionarily conserved and ligand-activated transcription factor involved in regulation of innate immune and a variety of cellular processes, including cell proliferation, differentiation, and death [21]. Typically, cytosolic AhR activated by hydrocarbons immediately translocate to the nucleus, dimerize with Arnt and generate AhR:Arnt complex, and bind to the elements of promoter region of target genes to activate gene expression [22]. As shown in Figure 4A, overexpression of both AhR and Arnt (p-AhR:Arnt) significantly enhanced the gga-miR-451 promoter activity, compared with p-AhR or p-Arnt. This suggested that the AhR:Arnt complex worked synergistically to activate the transcriptional expression of gga-miR-451 in MG infection. Recently, many target genes were reported to be induced by AhR and involved in diverse biological processes including immune responses, development, and hematopoiesis [23,24]. For instance, AhR regulates xenobiotic detoxification through induction of cytochrome P450 1A1 (CYP1A1) and activates immune responses in the intestine [25]. AhR induced the transcription factor Notch and was crucial for IL-22-production in gut ILC22 cells [26]. Moreover, we found AhR and Arnt were up-regulated in MG-HS infection, and AhR:Arnt decreased YWHAZ (14-3-3ζ ) expression probably through regulating gga-miR-451. It is reasonable to believe that AhR:Arnt transcription modulates gga-miR-451 expression following MG infection. In addition to AhR:Arnt, other transcription factors may also regulate miR-451 transcription. For example, c-Myc could bind on miR-451 promoter region and suppress miR-451⊣YWTAZ/AKT axis via recruiting HDAC3 in acute myeloid leukemia [27]. The upregulation of Paired box gene 4 (PAX4) directly regulates miR-451 expression and affects human epithelial cancer metastasis [28]. However, the molecular mechanism of these transcription factors that coordinate and regulate miR-451 gene expression needs to be further studied.

**Figure 7:**
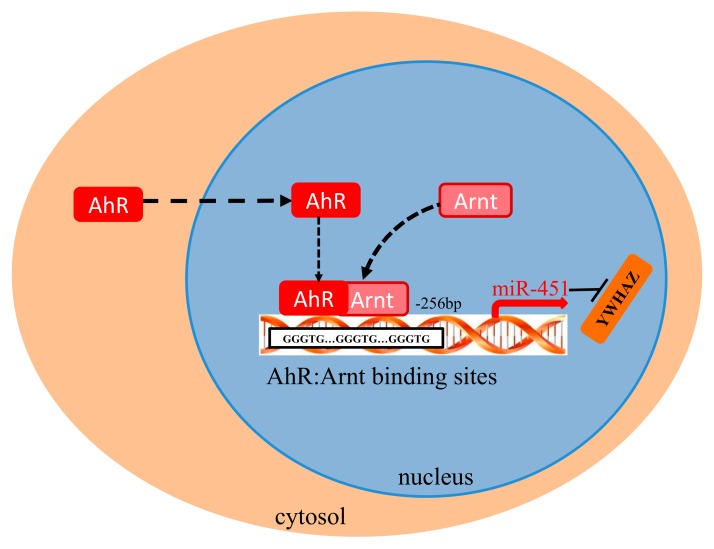
Graphical abstract showing the major findings of this study. Schematic summarizing the AhR:Arnt binds to the gga-miR-451 promoter and modulate gga-miR-451 transcription in DF-1 cells.

In conclusion, in the present study, we showed that the upregulation of gga-miR-451 is transcriptionally regulated in MG-infected DF-1 cells. A schematic presentation of the possible roles of AhR:Arnt in induction of gga-miR-451, as illustrated in Figure 7, showed that AhR:Arnt binds to the gga-miR-451 promoter and modulate gga-miR-451 transcription. Our discovery offers novel insight into the up-regulation of miR-451 and elucidates the mechanism of how the AhR:Arnt complex transcriptionally controls miR-451.

## 4. Materials and Methods

### 4.1. MG-HS Culture

The MG-HS strain used in this study is a virulent strain and donated by the State Key Laboratory of Agricultural Microbiology, College of Veterinary Medicine, Huazhong Agricultural University (Wuhan, Hubei, China). The strain was isolated from a chicken farm of Hubei Province in China [29,30]. The concentration determination of MG-HS and culture were performed as described previously. Color-changing unit (CCU) assay was used to determine the number of viable Mycoplasmas in a suspension; MG-HS at the mid-exponential phase (1 × 10^10^ CCU/mL) was used.

### 4.2. Cell Culture and Treatment

The chicken embryonic fibroblast cell line (DF-1) was purchased from ATCC® (Manassas, VA, USA), and maintained in Dulbecco’s modified Eagle medium (DMEM; Gibco, Carlsbad, CA, USA) containing 10% (*v/v*) FBS (Invitrogen, Carlsbad, CA, USA) and 1% penicillin/streptomycin (Gibco) antibiotics. For MG-HS infection experiments, cells were plated in 6-well plates and infected with 130 µL of MG-HS per well when the cells reached ∼80% confluence without antibiotics. For transfection experiments, cells were transfected with RNAs and/or plasmids using Lipofectamine 3000 (Invitrogen, Carlsbad, CA, USA) when the cells reached ∼70% confluence and were incubated for 36 h or 48 h, then harvested for further use.

### 4.3. Promoter Analysis

The gene-regulation software (http://gene-regulation.com) was used to predict the transcription factor binding sites in the promoters of gga-miR-451. Potential promoters were predicted by JASPAR (http://jaspar.genereg.net).

### 4.4. Plasmids Constructs

The full length of gga-miR-451 potential promoter region and 10 deletion fragments were amplified by PCR and sub-cloned into the *Kpn*I/*Hind*III sites of the pGL3-basic vector (Promega, Madison, WI, USA). Seven promoter constructs containing site-specific mutations were generated by overlap-expression PCR. To construct transcription factors AhR and Arnt expression vectors, CDS of AhR and Arnt were amplified from cDNA derived from DF-1 cells and cloned into pCDNA3.1 (p-AhR, p-Arnt). The expression vectors were double-digested with HindIII and *Kpn*I, and then cloned into the pCMV-C-HA vector (Beyotime, Shanghai, China). The recombinant plasmids were named pCMV-C-HA-AhR and pCMV-C-HA-Arnt, respectively. The primers are described in Table 1. All plasmids were verified by sequencing.

The sequences of all of the primers used in this study are shown in Table 1. RNA oligonucleotides were designed and synthesized by RIBOBIO (Guangdong, China) and are shown in Table 2.

### 4.5. Dual-Luciferase Reporter Assay

DF-1 cells in 24-well plates were separately co-transfected with the above plasmids (100 ng) and 10 ng of the Renilla luciferase expression vector pRL-TK (Promega, Madison, WI, USA) using Lipofectamine 3000 (Invitrogen, Carlsbad, CA, USA) for 12 h, and then either left uninfected or infected with 30 µL of MG-HS. At 24 hpi, the cells were harvested, and the detection of luciferase activity assay was performed using the dual-luciferase assay kit (Promega, USA), according to the manufacturer’s instructions. The results were normalized firefly luciferase activity to that of Renilla luciferase activity. All dual-luciferase reporter assays were performed in triplicate and repeated at least three times.

### 4.6. RNA Extraction and Quantitative Real-Time (RT-qPCR)

TRIzol reagent (Invitrogen, Carlsbad, USA) was used to extract the total RNA from treated DF-1 cells following to the manufacturer’s instructions. Reverse transcription was performed using the Prime Script^TM^ RT reagent kit with gDNA Eraser (TaKaRa, Tokyo, Japan). The expression levels of pMGA1.2, pri-miR-451, pre-miR-451, and AhR, Arnt were measured by RT-qPCR. RT-qPCR was performed on a CFX96 Touch^TM^ instrument (Bio-Rad, USA) using TransStart Top Green qPCR SuperMix (TRANSGEN, Beijing, China). The mRNA expression levels of related genes were standardized to Glyceraldehyde-3-phosphate dehydrogenase (GAPDH) expression using the 2^−ΔΔCt^ comparative method. The primers are listed in Table 1. Three independent repeats were performed for all above transfection experiments.

### 4.7. Chromatin Immunoprecipitation (ChIP)

ChIP was performed using the Magna ChIP A/C kit (Millipore, catalog no. 17-10086) according to the manufacturer’s instructions. In brief, 1 × 10^7^ DF-1 cells of 10-cm dishes were transfected with pCMV-C-HA-AhR:Arnt or pCMV-C-HA for 36 h, and then fixed with 1% formaldehyde for 10 min at room temperature. Subsequently, the lysates were sonicated and then immunoprecipitated with normal anti-mouse-IgG (Millipore, St. Louis, MO, USA) as a negative control or with the antibody against HA (Abcam, Cambridge, MA, USA, ab9110); 2 μg of antibody per 25 μg of DNA was used. The captured chromatin was eluted and un-cross-linked by heating at 62 °C, and then the immunoisolated DNA was subjected to qPCR amplification using primers (Table 1), TransStart Top Green qPCR SuperMix (TRANSGEN, China), and a real-time PCR detection system (Bio-Rad). The input DNA used for qPCR was equivalent to 0.01% of the original sample used for the ChIP assay.

### 4.8. Western Blot Analysis

12% SDS-PAGE were run and transferred onto polyvinylidene difluoride (PVDF) membranes (Millipore, USA). Then, 5% (*w/v*) fat-free milk was used to block membranes for 1 h at room temperature. Primary antibodies, including YWHAZ (Cat. D155211-0025, Sangon Biotech, Shanghai, China) and GAPDH (Cat.60004-1-1g, Proteintech, Wuhan, China), were incubated at 4 °C overnight, and goat anti-rabbit secondary antibodies were incubated at room temperature for 1 h. Washing with TBST was completed after each antibody incubation, and then the membranes were visualized with ECL™ detection system (Bio-Rad, Hercules, CA, USA).

### 4.9. Statistical Analysis

All experiments were performed in triplicate or higher. All results are presented as the mean ± SDs. Statistical significance was determined by either ANOVA for the comparison of multiple conditions or Student’s *t*-test for the comparison of two conditions, and significance was set at * *p* < 0.05, ** *p* < 0.01.

## Figures and Tables

**Table 1:** Sequences of DNA primers.

Name	Primer Sequence (5′-3′)	Accession No.
Primers for Cloning		
RP 2174-F	GGGTACCCTGGCTCTTGTCC	NC_006106.5
PR 1612-F	GGGTACCAGCAATGCTGTGACT	
PR 1147-F	GGGGTACCTGCTGCGATTTG	
PR 542-F	GGGGTACCAGTCTGGGATGAAG	
PR 386-F	GGGGTACCCATGCAGGTTCTAT	
PR 374-F	GGGTACCTATAAAGCGGGTGCAG	
PR 345-F	GGTACCACAAAGCAGCGGGGT	
PR 331-F	GGTACCTCACCCCGAGAGGTG	
PR 319-F	GGTACCCGAGAGGTGGGTGCT	
PR 254-F	GGGGTACCACACTAACGATGCTCT	
PR 177-F	GGGGTACCAGTTCACTATGAGACAC	
PR R	CCCAAGCTTAACGGTTTCCCTG	
Mut 1 F	GGTTCTATAAAGCATTCACAGCAGCAGCACCCCACAAA	
Mut 1 R	GCTGCTGCTGTGAATGCTTTATAGAACCTGCATGGCCTCATC	
Mut 2 F	CCCGAGAGGTTATGTCTGCCTACGGGGACGCAGC	
Mut 2 R	CCGTAGGCAGACATAACCTCTCGGGGTGACCCCG	
Mut 3 F	TTTGGGGACGAATGAACACTAACGATGCTCTCCTCTCCTC	
Mut 3 R	CATCGTTAGTGTTCATTCGTCCCCAAAGTGCCACCAC	
pcDNA3.1-AhR F	CAAGCTGGCTAGCGTTTAAACTTAAGCTTATGAACCCCAATGTCACCTAC	XM_025146991.1
pcDNA3.1-AhR R	CTGGACTAGTGGATCCGAGCTCGGTACCTTACATAAATCCACTAGATGCCAAATC	
pcDNA3.1-Arnt F	CAAGCTGGCTAGCGTTTAAACTTAAGCTTATGGCAGCCACCGCC	XM_025143392.1
pcDNA3.1-Arnt R	CTGGACTAGTGGATCCGAGCTCGGTACCTTACTCTGAAAAAGAAGGGAATATGT	
pCMV-HA-AhR F	TGGCGGCCGCTCTAGCCCGGGATGAACCCCAATGTC	XM_025146991.1
pCMV-HA-AhR R	GCGTAATCTGGAACATCGTATGGGTATCTAGACTCGAGTTACATAAATCCACTAGATGCC	
pCMV-HA-Arnt F	CAAGCTGGCTAGCGTTTAAACTTAAGCTTATGGCAGCCACCGCC	XM_025143392.1
pCMV-HA-Arnt R	CTGGACTAGTGGATCCGAGCTCGGTACCTTACTCTGAAAAAGAAGGGAATATGT	
Primers for RT-qPCR		
GAPDH-F	GAGGGTAGTGAAGGCTGCTG	NM_204305
GAPDH-R	CACAACACGGTTGCTGTATC	
pMGA1.2-F	TGAAACTTCGCTCAAAGAG	AF275312
pMGA1.2--R	TGTAACCCAACATCATCGT	
AhR-F	TTCCGCAGATTTCCTCCCAC	XM_025146991.1
AhR-R	GCTGAGCCTAAGCACAGACA	
Arnt-F	AAACTGGAGGAGGCATCGTG	XM_025143392.1
Arnt-R	GTCAGCTTGTCTGGTTTGCG	
gga-miR-451-F	GTAGGAAACCGTTACCATTACTGAG	MIMAT0003775
gga-miR-451-R	ACTGGTGTCGTGGAGTCGGC	
gga-5s-rRNA-F	CCATACCACCCTGGAAACGC	
gga-5s-rRNA-R ChIP 141bp F ChIP 141bp R	TACTAACCGAGCCCGACCCT CCATGCAGGTTCTATAAAGCG GGAGAGCATCGTTAGTGTCAC	NC_006106.5

**Table 2:** Sequences of RNA oligonucleotides.

Name	Sequences (5′-3′)
si-AhR	CCAACTTCCTCCAGAGAAT
si-Arnt	CAGACAAGCTGACCATCTT

## Supplementary Materials

The following are available online at https://www.mdpi.com/1422-0067/20/12/3087/s1.
