# Supplementary material for: Transcriptional Regulation of gga-miR-451 by AhR:Arnt in Mycoplasma gallisepticum (HS Strain) Infection
Source: Int J Mol Sci. 2019 Jun 24;20(12):3087. doi: 10.3390/ijms20123087 (PMC6627052; doi:10.3390/ijms20123087)
Supplement: Supplementary file 1 [file ijms-20-03087-s001.pdf]

Figure S1

A

V$YY1_02	2	(+)	1.000	0.849	atgagGCCATgcaggttcta	YY1	
V$CAAT_01	3	(+)	0.837	0.843	tgaggCCATGca	CCAAT box	
V$AREB6_01	10	(-)	1.000	0.907	atgCAGGTtctat	AREB6	
V$TATA_C	16	(-)	0.841	0.804	gTTCTAtaaa	TATA	
V$BARBIE_01	19	(+)	1.000	0.907	ctatAAAGCgggtgc	Barbie Box	
V$AHRARNT_01	20	(+)	0.820	0.805	tataaagcGGGTGcag	AhR/Arnt	2
V$USF_Q6	25	(-)	0.886	0.828	agcgGGTGCa	USF	
V$SREBP1_02	39	(+)	0.800	0.887	caGCACCccac	SREBP-1	
V$AHRARNT_01	39	(-)	0.820	0.802	cagCACCCcacaaagc	AhR/Arnt	2
V$BARBIE_01	46	(+)	1.000	0.918	ccacAAAGCagcggg	Barbie Box	
V$VMAF_01	52	(-)	0.870	0.805	agcagcggggTCACCcga	v-Maf	
V$RORA1_01	53	(+)	1.000	0.870	gcagcggGGTCAc	RORalpha1	
V$AP1_Q2	56	(-)	0.962	0.935	gcggGGTCAcc	AP-1	
V$AP1_Q4	56	(-)	0.935	0.897	gcggGGTCAcc	AP-1	
V$CREB_02	57	(-)	0.820	0.819	cggGGTCAcccc	CREB	

B

Matrix ID	Name	Score	Relative score	Sequence ID	Start	End	Strand
MA0006.1	Ahr::Arnt	5.29689	0.817304689639	1	9	14	-
Predicted sequence TGCATG							
MA0006.1	Ahr::Arnt	5.29689	0.817304689639	1	75	80	+
Predicted sequence TGGGTG							
MA0006.1	Ahr::Arnt	5.23853	0.81482862249	1	92	97	-
Predicted sequence TGCGTC							
MA0006.1	Ahr::Arnt	4.89067	0.800069129061	1	27	32	+
Predicted sequence CGGGTG							

Figure S1 Bioinformatics analysis of the potential transcription factors in gga-miR-451. (A) The prediction from Gene-regulation. (B) The prediction from JASPAR.

Figure S2

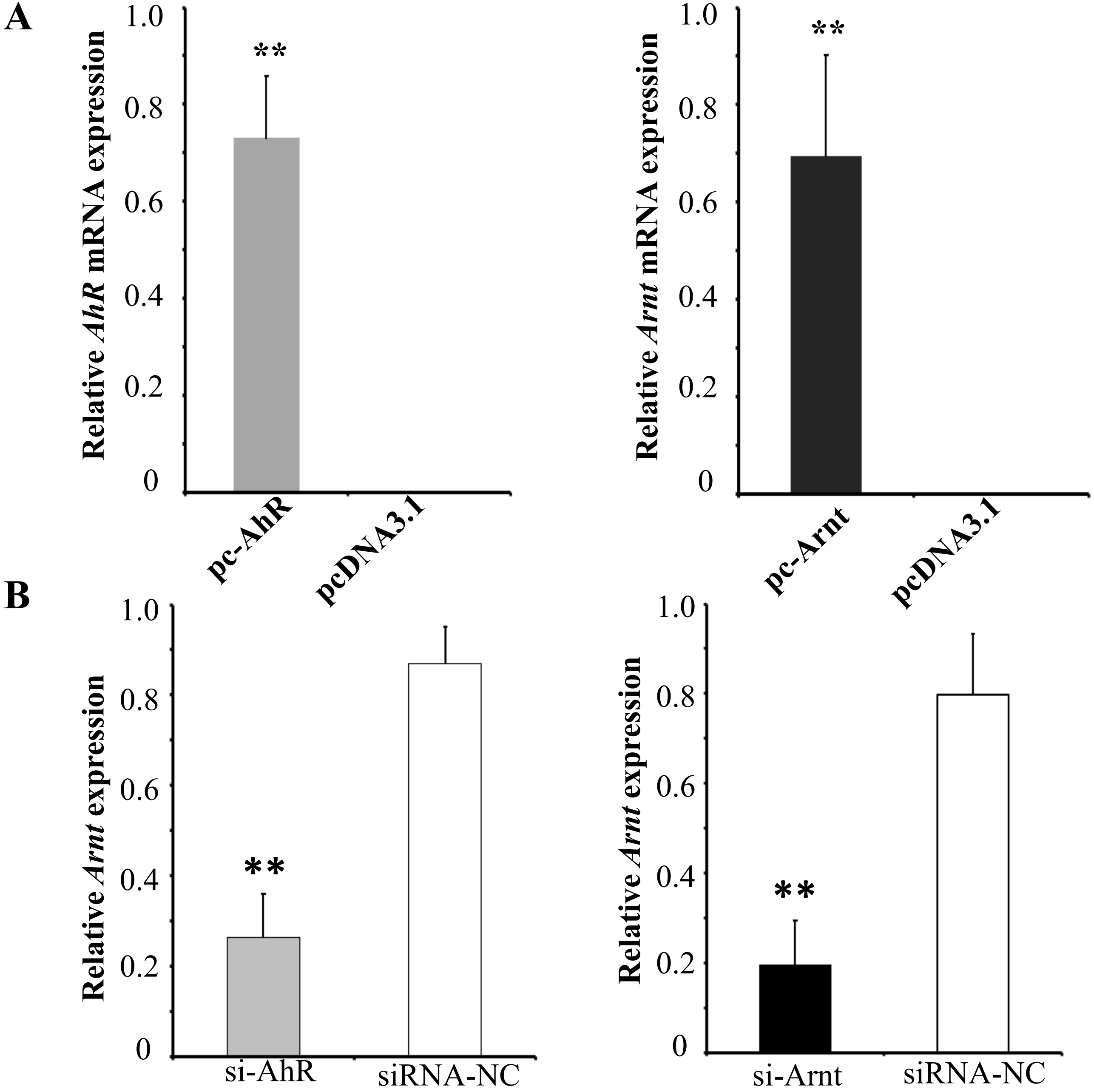

Figure S2 Overexpression and silence of AhR and Arnt. (A) DF-1 cells were transfected with pcDNA3.1-AhR (p-AhR), pcDNA3.1-Arnt (p-Arnt) or pcDNA3.1 for 48 h. **(B)** DF-1 cells were transfected with siRNA-AhR (si-AhR), siRNA-Arnt (si-Arnt) or siRNA NC for 48 h. AhR and Arnt mRNA levels were measured by RT-qPCR. The expression of GAPDH was used as a loading control.
